# Comparison Effects of Ruminal Crabtree-Negative Yeasts and Crabtree-Positive Yeasts for Improving Ensiled Rice Straw Quality and Ruminal Digestion Using In Vitro Gas Production

**DOI:** 10.3390/jof6030109

**Published:** 2020-07-15

**Authors:** Chanon Suntara, Anusorn Cherdthong, Suthipong Uriyapongson, Metha Wanapat, Pin Chanjula

**Affiliations:** 1 Tropical Feed Resources Research and Development Center (TROFREC), Khon Kaen 40002, Thailand; Chanon_su@kkumail.com (C.S.); metha@kku.ac.th (M.W.); 2 Department of Animal Science, Faculty of Agriculture, Khon Kaen University, Khon Kaen 40002, Thailand; suthipng@kku.ac.th; 3 Department of Animal Science, Faculty of Natural Resources, Prince of Songkla University, Songkhla 90112, Thailand; pin.c@psu.ac.th

**Keywords:** Crabtree-negative ruminal yeast, Crabtree-positive yeast, ensiled rice straw, in vitro fermentation

## Abstract

The objective of this study was to compare the effects of Crabtree-negative ruminal yeast and Crabtree-positive yeast in ensiled rice straw (RS) on the ensilage quality, nutritive value, and microorganism composition, including the evaluation of the ensiled RS using the in vitro gas production technique. The experiment was conducted in a 4 × 3 factorial arrangement in a randomized complete design. Factor A was yeast species with no inoculant, Crabtree-negative yeasts (*Pichia kudriavzevii* KKU20 and *Candida tropicalis* KKU20), and Crabtree-positive yeast (*Saccharomyces cerevisae*), whereas factor B was ensilage times (7, 14, and 21 days). The rate of growth was revealed to be lower in Crabtree-positive yeasts than the other Crabtree-negative yeast strains (*p* < 0.01). RS ensiled with *S. cerevisiae* showed decreased dry matter (DM) content by 9.0% when compared to the sample without a yeast inoculant. In addition, organic matter (OM) content was greater (*p* < 0.01) for *P. kudriavzevii* KKU20 than *C. tropicalis* KKU20 and without an inoculant. Neutral detergent fiber (NDF) content was significantly decreased (*p* < 0.01) by yeast inoculants by about 2.75% when compared to the control group. Lactic acid bacteria (LAB) and aerobic bacteria were low (*p* < 0.05) when yeasts were added. However, no interaction was found between yeast and ensilage times on the quality of ensiled RS (*p* > 0.05). The *P. kudriavzevii* KKU20 addition was associated with the highest value (*p* < 0.01) of gas produced—an insoluble fraction (b), potential extent of gas production (a + b), and cumulative gas production at 96 h—when compared with *S. cerevisiae* or the control group. The highest in vitro dry matter digestibility and in vitro neutral detergent fiber digestibility was observed in RS ensiled with *P. kudriavzevii* KKU20 for 14 days (*p* < 0.01, *p* < 0.05). The maximum total volatile fatty acids (VFAs) at 4 and 8 h of incubation and the mean value were observed in RS ensiled with *P. kudriavzevii* KKU20 (*p* < 0.01). RS ensiled with all yeast strains showed an increased propionate concentration at 8 h (*p* < 0.01). In conclusion, ensiling RS with isolated Crabtree-negative ruminal yeasts could benefit feed digestion and in vitro gas production more than Crabtree-positive yeast does. *P. kudriavzevii* KKU20, an isolated Crabtree-negative ruminal yeast used to treat RS, had the highest potential for increasing cumulative gas production and enhancing in vitro digestibility.

## 1. Introduction

Going back several years, yeast inoculants have been confirmed to improve the efficiency of feed utilization and the performance of ruminants [1]. The nutritional value of feedstuffs and byproducts can be improved through fermentation with yeast [2]. In tropical regions, rice straw (RS) is an abundant byproduct, but its nutrient levels are low, especially levels of crude protein (CP) [3]. To improve RS nutrition, yeast was chosen as an alternative solution for providing high-quality protein, vitamins, stimulatory factors, and positive functions to improve the rumen fermentation process. An earlier work by Foiklang et al. [4] demonstrated that *Saccharomyces cerevisiae* (*S. cerevisiae*)-fermented RS showed enhanced gas production at 96 h and that true digestibility at 48 h increased 220 g/kg and 96 g/kg, respectively.

Although *S. cerevisiae* has become the model organism used for improving animal performance [5,6] in the past two decades, the most recent study indicated that *S. cerevisiae* is limited to producing cell biomass during glucose fermentation in aerated conditions. De Deken [7] was the first to report that *S. cerevisiae* exhibits alcoholic fermentation instead of biomass production, thus resulting in the production of little biomass. This phenomenon is called the Crabtree effect, and yeasts expressing this trait are called “Crabtree-positive yeasts” [8]. Van Urk et al. [9] reported that *S. cerevisiae* had low potential for proliferation under excessive glucose and even aerobic conditions. In addition, Wardrop et al. [10] found that *S. cerevisiae* provides 7-fold lower biomass compared to other strains when cultured in a solution medium with excessive glucose. These may lead to lower microbial protein contents, thus causing underperformance with regard to animal feed with Crabtree-positive yeasts. Therefore, the potential yeast produces high biomass, and some specific enzymes might be further synthesized and elucidated.

Nevertheless, some yeasts do not present the Crabtree effect and, consequently, these yeasts might produce greater biomass in aerobic culture than Crabtree-positive yeasts; these are called “Crabtree-negative yeasts” [10]. Crabtree-negative yeasts might be the most interesting option, as they have the special characteristic of limited fermentative products, and biomass and carbon dioxide are the sole products under aerobic conditions [8]. This phenomenon is very important because it introduces the opportunity to choose high-potential yeasts that can produce more biomass and can be applied in animal feed [11]. High yeast biomass may supply essential rumen fermentation factors and be a greatly nutritious feed (protein, amino acids, and vitamins) supplement for ruminants. Thus, it could enhance performance production and greater health in a cost-effective manner. Aside from biomass production, another benefit of using yeast is studying fiber digestibility. A previous study using yeast fermented with cassava pulp saw an improvement in nutritional value but not in fiber content [2]. Therefore, the discovery of a yeastproduced cellulase enzyme may enhance the utilization of animal feed. Recently, Suntara and Cherdthong [12] isolated 10 yeast strains from rumen fluids and identified 2 high-potential strains that were classified as Crabtree-negative yeasts, namely *Pichia kudriavzevii* KKU20 (*P. kudriavzevii* KKU20) and *Candida tropicalis* KKU20 (*C. tropicalis* KKU20). These ruminal yeasts can produce high biomass and a cellulase enzyme. Unfortunately, the capability of yeast to digest fiber has never been used to improve the utilization of feedstuff; however, previous experiments that isolated yeast from the rumen made it possible to improve the nutritive value of ensiled RS or to digest the fiber content during the ensilage process [12]. It was hypothesized that the Crabtree-negative ruminal yeasts *P. kudriavzevii* KKU20and *C. tropicalis* KKU20 could beneficially affect RS and its utilization.

Therefore, the objective of this study was to compare the effects of Crabtree-negative ruminal yeast and Crabtree-positive yeast in ensiled RS on the ensilage quality, nutritive value, and microorganism composition, including the evaluation of the ensiled RS using the in vitro gas production technique.

## 2. Materials and Methods

Animals involved in this study were approved by the Animal Ethics Committee of Khon Kaen University (record no. IACUC-KKU 38/62), based on the Ethics of Animal Experimentation of National Research Council of Thailand.

### 2.1. Yeast-Fermented Rice Straw, and Experimental Design

The current study was conducted at the Department of Animal Science, Faculty of Agriculture, Khon Kaen University, Khon Kaen, Thailand, from June 2018 to September 2018. RS (*Oryza sativa* L.) was taken from the Tropical Feed Resources Research and Development Center (TROFREC) at Khon Kaen University, and RS was chopped by machine (ET110, Kubota engine Co., Ltd., Chachoengsao, Thailand) into lengths of 5–10 cm. Crabtree-negative ruminal yeasts included *Pichia kudriavzevii* KKU20 (*P. kudriavzevii* KKU20) and *Candida tropicalis* KKU20 (*C. tropicalis* KKU20) with strain numbers CBS 5147^T^ (MH545928) and CBS 94^T^ (U45749), and they were obtained by isolating, screening, and identifying the rumen of crossbred Thai–Holstein Friesian dairy cattle [12]. These ruminal yeasts were tested for their high-potential biomass (17.07 g/L) and cellulase enzymes (0.075 unit/mL) production. Crabtree-positive yeast, *S. cerevisae*, was obtained from baker’s yeast (Perfect yeast Co., Ltd., Ubon Ratchathani, Thailand). The method of producing ensiled RS was divided into three steps: first, yeasts were cultured and amplified using 250 g/kg sugarcane molasses with a 10 g/kg urea solution, and then the solution’s pH was adjusted using formic acid (L.C. industrial Co., Ltd., Nakhon Pathom, Thailand) to achieve a final pH of 3.5 [2]; second, an oxygen flush was used to determine what the yeasts required to complete respiration for cell growth; and lastly, mixed-media solution was applied to the RS and then ensiled in a plastic bag (E-Sann Pass Pack 1999 Co., Ltd., Khon Kaen, Thailand), with five replications for each ensilage according to Reference [4]. The experiment was conducted in a 4 × 3 factorial arrangement in a randomized complete design. Factor A was yeast species, with no inoculant, Crabtree-negative yeasts (*P. kudriavzevii* KKU20 and *C. tropicalis* KKU20), and Crabtree-positive yeast (*S. cerevisiae*), whereas factor B was ensilage time (7, 14, and 21 days). One hundred gram samples of mixed RS were packed into plastic bags (size 8 × 12 cm), sealed with a vacuum sealer (DZ-400 vacuum machine, Nakhoncenterpack Co., Ltd., Nakhon Ratchasima, Thailand), and ensiled at room temperature. Silos of all ensiled RS samples were exposed after 7, 14, and 21 days and were sampled to determine the fermentation products and nutritional composition.

### 2.2. Fermentation Products and Chemical Composition

For silage analysis, samples were dried at lower temperatures (60 °C) in an oven for 48 h and ground by forcing them through a 1 mm steel screen (Wiley mill, Arthur H. Thomas Co., Philadelphia, PA, USA) prior to chemical analysis. Ensiled RS samples were measured for dry matter (DM; ID 967.03), ash (ID 492.05), ether extract (EE; ID 455.08), and CP (CP; ID 984.13), according to the method of Reference [13]. Neutral detergent fiber (NDF) and acid detergent fiber (ADF) content were determined using a detergent analysis method [14], acid detergent lignin (ADL), according to Van Soest [15]. Ensiled RS was extracted using cold water and determinations were made according to Cai [16]. Fresh silage (10 g) was mixed with 90 mL of sterilized water and kept at 4 °C [17]. The solution’s pH was determined using a glass electrode pH meter (Hanna HI-8424 Portable pH/ORP Meter, Woonsocket, RI, USA).

Subsamples of silage fluid were centrifuged at 16,000 rpm for 15 min, and the liquid above the solid residue was filtered using a 0.45 micron syringe filter. Lactic acid (LA), acetic acid (C_2_), propionic acid (C_3_), and butyric acid (C_4_) analyses were performed using a high-performance liquid chromatography (HPLC) machine (Shimadzu LC-20A, Kyoto, Japan) equipped with an Inertsil ODS-3 C18 (250 mm × 4.6 mm i.d., 5 µm) column and mobile phase: phosphoric acid 25 mM, flow rate: 1 mL/minute, detection (UV): 210 nm: Injection: 20 microliters, according to Prueksatrakul et al. [18]. Ammonia–nitrogen (NH_3_–N) concentration was determined according to the Kjeldahl methods [13].

### 2.3. Yeast Counts and Microorganism Analysis of Ensiled RS

The total amount of yeast-enriched solution medium before RS ensilage was determined in order to calculate the cell growth pattern, which was expressed in the form of total cell/mL by direct count using a hemocytometer under a microscope [19]. Microorganism populations, including yeast, lactic acid bacteria (LAB), coliforms, aerobic bacteria, and mold after ensilage of RS were determined by counting the individual colonies according to Kozaki et al. [20], and viable colonies were reported as colony-forming units (cfu) per gram of fresh matter (cfu/g FM). Ten grams of ensiled RS was shaken with 90 mL of sterilized, distilled water, and serial dilutions in 85 g of sodium chloride in distilled water (e.g., 1 L of distilled water at 10^−1^, 10^−3^, and 10^−5^) were performed. Measures of 20 µL of various solutions were then spread on agar plates. Yeast and mold were cultivated on potato dextrose agar (Sisco Research Laboratories Pvt. Ltd., Maharashtra, India); yeast was distinguishable from molds and microbes based on observations of colony presence and cell morphology. The LAB was counted on Lactobacilli MRS agar (Sisco Research Laboratories Pvt. Ltd., Maharashtra, India) in a box without oxygen at 30 °C for 48 h (Sugiyamagen Ltd., Tokyo, Japan). The presence of coliforms was detected on a blue light broth agar plate (Nissui-Seiyaku Co., Ltd., Tokyo, Japan). Bacilli and aerobic microbes were cultivated on nutrient agar plates under anaerobic conditions for 24 h at 30 °C. Incubated plates were inverted at 30 °C for 2 days, and the mold was counted.

### 2.4. In Vitro Gas Production and Digestibility

Rumen liquor was collected from two rumen-fistulated dairy cattle with body weights of 350 ± 30 kg. The animals were kept in separate pens and ensiled RS was fed *ad libitum* along with a concentrated diet (160 g/kg CP) according to the National Research Council (NRC) [21] requirement for dairy cattle, which included supplementing with a suitable mineral lick and permanent access to a clean water supply for at least 14 days. Samples were collected through a rumen cannula on Day 15, when 1000 mL of rumen fluid was taken from the cattle via cannula before morning feeding. The samples were filtered through a cheesecloth folded to form four layers and into a bottle with thermal insulation (39 °C), and then they were transported to the laboratory within 15 min. Rumen medium preparations containing distilled water (1095 mL), a micromineral mixture (0.23 mL), a macromineral mixture (365 mL), a resazurin mixture (1 mL), a reduction mixture (60 mL), and a buffer mixture (730 mL) were blended with the rumen liquor (660 mL) in non-oxygen conditions.

The ground ensiled RS of 0.2 g and concentrated diet of 0.3 g (160 g/kg of CP and 720 g/kg of total digestible nutrient (TDN)) were added to 50 mL bottles (three replications per sample). The dietary treatments were tested in triplicate within the incubation, and incubations were repeated on 3 separate days (runs). Butyl rubber stoppers and aluminum caps provided a gas-tight seal for all laboratory bottles. Next, 40 mL of rumen liquor medium was added to each treatment bottle using an 18 gauge × 1.5 inch needle. Treatment bottles were incubated in a hot-air oven at 39 °C for 96 h to test gas-production kinetics. Every 3 h of incubation, the bottles were gently shaken, and three treated bottles and three untreated bottles were included in each run. The untreated bottles (blank) contained only rumen liquor, and the average value of the gas yields from these bottles was subtracted from the treated bottles to determine net gas production. Gas production was measured using a 25 mL calibrated syringe. The bottle was punctured using an 18 gauge injection needle placed in the heating chamber. During incubation, the production of rumen gas patterns was evaluated at 0, 0.5, 1, 2, 4, 6, 8, 12, 18, 24, 48, 72, and 96 h, and was noted as the gas yield amount according to Kozaki et al. [22].

At 48 h, the fermented residues were filtered into an Ankom filter bag (ANKOM 200, ANKOM Technology, New York, NY, USA), dried at 60 °C in an oven for 72 h, and assessed for dry matter digestibility (IVDMD). The dried feed sample and remaining residue were ashed at 550 °C for the determination of in vitro organic matter degradability (IVOMD) [23]. Dried residues were extracted with an NDF and ADF solution to determine in vitro NDF digestibility (IVNDFD), in vitro ADF digestibility (IVADFD), and in vitro true digestibility (IVTD) according to Van Soest et al. [14].

### 2.5. In Vitro Ruminal Fermentation and Ruminal Microbial Counts

Rumen liquor was sampled from 144 bottles at 2 and 4 h after incubation (72 samples were sampled each time) to determine its pH. Afterward, samples were filtered through cheesecloth and centrifuged at 16,000× *g* for 15 min. The liquid above the solid residue used for NH_3_–N analysis was measured quantitatively according to Association of Official Analytical Chemists (AOAC) [13] and volatile fatty acids (VFAs) were measured using the HPLC machine (Shimadzu LC-20A, Kyoto, Japan) equipped with an Inertsil ODS-3 C18 (250 mm × 4.6 mm i.d., 5 µm) column and mobile phase: phosphoric acid 25 mM, flow rate: 1 mL/minute, detection (UV): 210 nm: Injection: 20 microliters [24]. After incubation for 2 and 4 h, the liquid in the serum bottles was blended well, and a 1 mL sample was incorporated with 9 mL of formaldehyde to evaluate the rumen’s microbes. Ruminal fungal zoospore, protozoa, and bacterial populations were measured using a manual counting method and a hemocytometer (Boeco, Hamburg, Germany).

### 2.6. Statistical Analysis and Calculation

The model of Ørskov and McDonald [25] was used for cumulative gas production curves.

Y = a + b (1 − e^(−ct)^) (1)

where a = volume of gas produced from soluble fraction, b = volume of gas produced from insoluble fraction, c = gas production rate constant for insoluble fraction, t = incubation time, a + b = potential extent of gas production, and Y = gas produced at time t.

Data for silage quality, chemical composition, microorganism counts, in vitro digestibility, and gas kinetics of ensiled RS were examined. Data were measured in a completely randomized design with a 4 × 3 (yeast species × ensilage times) factorial treatment by ANOVA using general linear model (GLM) procedures [26]. The average value of treatment was computed using the least-square means (LSMEANS) option of SAS, with statistical modeling as follows:

Yij = μ + Ai + Bj + ABij + εij(2)

where Yij = observation, μ = overall mean, Ai = yeast species effect (i = no inoculant, *P. kudriavzevii* KKU20, *C. tropicalis* KKU20 and *S. cerevisiae*), Bj = ensilage time effect (j = 7, 14 and 21 days), ABij = yeast species effect × ensilage time effect, and εij = error. When F-tests were significant, single degree of freedom orthogonal contrasts were used to determine the contrast between factors. Duncan’s new multiple-range test (DMRT) was used to determine the differences mean of treatments at *p <* 0.05 [27].

## 3. Results

### 3.1. Viable Cell Counts in Medium Solution of Crabtree-Negative Ruminal Yeast and Positive Yeasts

**Figure 1:**
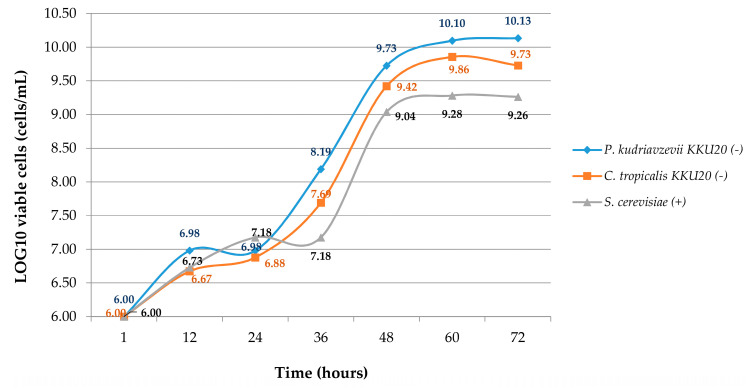
Cell growth pattern of three selected yeasts strains (Crabtree-positive and -negative yeasts) expressed under aerobic condition in molasses 250 g/kg with urea 10 g/kg by viable direct count.

Growth curve experiments showed that different species of yeast grew well in 250 g/kg of sugarcane molasses with 10 g/kg of urea (Figure 1). The viable cell count in a medium solution started from 6.0 Log10 cell/mL, and the growth of *P. kudriavzevii* KKU20, *C. tropicalis* KKU20, and *S. cerevisiae* was 10.13, 9.73, and 9.26 Log10 cell/mL at 72 h of incubation, respectively. The exponential growth phase of yeast was detected between 36 and 60 h after being cultured in a medium solution. Yeasts’ cell growth remained continually stable over the 60 h of incubation. The rate of growth was revealed to be lower in *S. cerevisiae* (Crabtree-positive yeast) than in the other strain (Crabtree-negative yeast) (*p* < 0.01). At 72 h, the maximum growth rate was observed in 10.13 Log10 cell/mL of *P. kudriavzevii* KKU20, whereas the growth rates of *C. tropicalis* KKU20, and *S. cerevisiae* were found to be lower at 9.73 and 9.26 Log10 cell/mL, respectively (*p* < 0.01).

### 3.2. Chemical Compositions of Rice Straw Ensiled with Yeast

Table 1 shows the chemical compositions of RS ensiled with Crabtree-positive and Crabtree-negative yeasts. The contents of EE, CP, ADF, ADL, hemicellulose, and cellulose were not affected (*p* > 0.05) by yeast strain. There were no interactions (*p* > 0.05) between the yeast strain and ensilage times for DM, whereas RS ensiled with *S. cerevisiae* decreased DM content by 9.0% when compared to the sample without a yeast inoculant. Moreover, ensilage at 21 days decreased (*p* < 0.01) the DM content of RS. OM content was greater *(p* < 0.01) for *P. kudriavzevii* KKU20 than *C. tropicalis* KKU20 and without an inoculant, whereas ensilage times up to 7 days provided the greatest (*p* < 0.05) OM content in the fermented RS. NDF content was significantly decreased (*p* < 0.01) by yeast inoculants by about 2.75% when compared to the control group.

### 3.3. Microbiological Analysis of Ensiled RS

Interactions were observed between yeast strains and ensilage times for the yeast population and aerobic bacteria count (*p* < 0.05, Table 2). The yeast population was highest (*p* < 0.05) in RS ensiled with *P. kudriavzevii* KKU20 at 7 and 14 days (10.3 and 9.44 Log10 cfu/g FM), *C. tropicalis* KKU20 at 7 days (9.85 Log10 cfu/g FM), and *S. cerevisiae* at 7 and 14 days (10.1 and 9.56 Log10 cfu/g FM). Nevertheless, aerobic bacteria were lowest (*p* < 0.05) in RS ensiled with *P. kudriavzevii* KKU20 or *S. cerevisiae* for 7 days (2.98 Log10 cfu/g FM and 2.90 Log10 cfu/g FM, respectively). Moreover, the yeast colony count in ensiled RS was higher (*p* < 0.05) than in the control (9.03 vs. 4.85 Log10 cfu/g FM), whereas LAB and aerobic bacteria were low (*p* < 0.05) when yeasts were added (6.90 vs. 5.26 and 6.27 vs. 4.39 Log10 cfu/g FM, respectively).

### 3.4. Ensilage Quality of Ensiled RS

No interaction was found between yeast and ensilage times for the quality of ensiled RS (*p* > 0.05). However, the yeast strain influenced LA and C_2_, whereas the pH was affected by ensilage time (*p* < 0.01) (Table 3). The LA decreased by about 11.9%, whereas C_2_ increased by about 24.2% (*p* < 0.01) when yeasts were added to RS. The pH value increased (*p* < 0.01) in ensiled RS for 21 days. In addition, there were no changes (*p* > 0.05) in the concentrations of NH_3_–N or C_4_.

### 3.5. In Vitro Gas Production and Degradability

An interaction between yeast stains and ensilage times was observed for the gas production rate constant for the insoluble fraction (c value) (*p* < 0.01) and IVDMD (*p* < 0.01) and IVNDFD (*p* < 0.05). The volume of gas produced from an insoluble fraction (b value), potential extent of gas production (a + b), and cumulative gas production at 96 h were affected by yeast strains, but the volume of gas produced from a soluble fraction (a value), which did not change (*p* > 0.05) (Table 4). The *P. kudriavzevii* KKU20 addition was associated with the highest value (*p* < 0.01) of b, a + b, and cumulative gas production at 96 h (122.8, 127.4, and 123.6 mL/0.5 g DM substrate, respectively) when compared with *S. cerevisiae* or the control group. RS fermented with *P. kudriavzevii* KKU20 for 14 to 21 days showed the highest c value, whereas *C. tropicalis* KKU20 showed the highest c value for 7, 14, and 21 days (*p* < 0.01).

RS ensiled with yeast strains showed significantly higher (*p* < 0.01) degradability of IVDMD, IVOMD, IVNDFD, and IVADFD than the control, which ranged from 15.1%, 14.7%, 3.6%, and 6.4%, respectively. The highest IVDMD and IVNDFD were observed in RS ensiled with *P. kudriavzevii* KKU20 for 14 days at 639.8 and 774.0 g/kg DM, respectively (*p* < 0.01, *p* < 0.05).

### 3.6. In Vitro Ruminal Volatile Fatty Acids

The yeast strains influenced the concentrations of total VFAs (TVFAs), C_2_, C_3_, and C_4_ when ensiled RS was used as a substrate (*p* < 0.01) (Table 5). The maximum TVFAs at 4 and 8 h of incubation and the mean value were observed in RS ensiled with *P. kudriavzevii* KKU20 (*p* < 0.01) for 88.52, 89.7, and 89.1 mmol/L, respectively. At 8 h of incubation, RS fermented with yeast caused a decrease of C_2_ in rumen fluids of approximately 8.86% when compared with the control, whereas the mean value of C_2_ showed the lowest value when RS was fermented with *P. kudriavzevii* KKU20 for 14 days (64.2 mol/100 mol, *p* < 0.05). RS ensiled with all yeast strains showed an increased C_3_ concentration at 8 h and mean values of 24.8% and 22.9%, respectively, whereas the control group was about 17.3% and 16.8% (*p* < 0.01). At 4 h of incubation, the concentration of C_4_ in rumen fluids from RS ensiled with all yeast strains was 9.58%, which was lower than the control at 10.8% (*p* < 0.01). At 8 h of incubation, the concentration of C_4_ showed the lowest value (8.3 mol/100 mol) when the RS was fermented with *C. tropicalis* KKU20 for 21 days (*p* < 0.01), whereas the mean value was lowest (8.46 mol/100 mol) when the RS was fermented with *P. kudriavzevii* KKU20 for 14 days (*p* < 0.01).

### 3.7. Microbial Populations in Rumen Fluids

Regarding ensiled RS, populations of bacteria, protozoa, and fungal zoospores of incubation fluids were affected (*p* < 0.01) by yeast species (Table 6). No interaction effects (*p* > 0.05) existed between yeast strains and ensilage times on a bacterial population at 4 h. However, the RS ensiled with *P. kudriavzevii* KKU20 showed a higher (*p* < 0.01) bacterial population when compared with other strains. However, at 21 days of ensiled RS, the bacterial population decreased 4.2% in rumen fluids (*p* < 0.01). There were interactions between yeast strain and ensilage time on bacterial populations at 8 h and the mean value (*p* < 0.01). The RS ensiled with *P. kudriavzevii* KKU20 for 7, 14, and 21 days shows the highest bacterial population at approximately 8.29, 8.27, and 8.22 Log10 cell/mL, respectively. *C. tropicalis* KKU20 fermented with RS for 7, 14, and 21 days showed the highest bacterial populations at approximately 8.24, 8.26, and 8.28 Log10 cell/mL, respectively (*p* < 0.01).

Compared to Crabtree-positive yeast, the bacterial populations at 4 and 8 h of incubation and the mean value increased by 3.1%, 3.9%, and 3.4%, respectively, in RS ensiled with Crabtree-negative ruminal yeast. No interactions (*p* > 0.05) were observed for protozoal populations; however, *S. cerevisiae* addition decreased (*p* < 0.01) the protozoal population at 8 h and the mean value by 7.2% and 4.9%, respectively. Fungal zoospores at 4 and 8 h and the mean value increased (*p* < 0.01) by 10.3%, 10.1%, and 10.1%, respectively, when yeasts were added compared to the inoculant group with no yeast.

## 4. Discussion

### 4.1. Chemical Compositions Changed in Ensiled RS

In the present study, concerning a medium solution under aerobic conditions and a high concentration of sugarcane molasses, Crabtree-negative yeast showed high growth potential when compared to Crabtree-positive yeast. Typically, sugar can be converted into yeast cells and then to a pyruvate, as well as moving into the mitochondria through the activity of the pyruvate dehydrogenase complex (PDH complex). Sufficient oxygen supports the tricarboxylic acid cycle (TCA cycle) and the electron-transport chain (ETS) to complete their processes, and yeast can then produce maximum adenosine triphosphate (ATP) [28]. However, the metabolism was different with regard to different species of yeast, namely Crabtree-negative and Crabtree-positive yeasts. High-sugar conditions (glucose around 100–200 mg/L) are a key factor inhibiting the PDH complex enzyme [8], whereas it activates the pyruvate decarboxylase 3–4-fold and changes sugar to ethanol (as well as sufficient oxygen) in a Crabtree-positive yeast like *S. cerevisiae* [29,30]. Meanwhile, for Crabtree-negative yeasts (*P. kudriavzevii* KKU20 and *C. tropicalis* KKU20), the pyruvate was used in another channel for conversion to biomass (not ethanol). Pronk et al. [28] stated that the pyruvate dehydrogenase bypass (PDH bypass) is the main pathway by which sugar converts to pyruvate and acetate before transforming to cytosolic acetyl CoA by means of the carnitine acetyltransferase system, and moves to the mitochondria later on. The different pathways allow differing end products, which could result in the fact that Crabtree-negative yeast provides more biomass than Crabtree-positive yeast.

These phenomena are in agreement with Van Urk et al. [9], who reported that the growth rates of Crabtree-positive yeasts did not increase under aerobic conditions with added glucose. Moreover, Wardrop et al. [10] studied the effect of glucose levels from 1 to 50 g/L on the biomass conversion of *S. cerevisiae* (Crabtree-positive yeast) and *K. marxianus* (Crabtree-negative yeast), and the data showed that *K. marxianus* provided 7 times more biomass than *S. cerevisiae,* which predominantly produced ethanol. In the present study, 250 g/kg of sugarcane molasses was used as a carbon source, which supplies a high amount of sugar and may inhibit the PDH complex in *S. cerevisiae*. Sugarcane molasses consists of 90 g/kg of glucose [31]; thus, glucose levels were around 22.5 g/L in this study and influenced *S. cerevisiae*.

The reduction of DM was observed in RS ensiled for 21 days with *S. cerevisiae*. DM loss can be caused by many factors, one of which is the effect of ethanol. High ethanol content is associated with DM loss because it can increase the temperature in silage during fermentation [32]. Furthermore, the DM loss observed in the *S. cerevisiae* treatment compared to that of the control might have been a fermentative DM loss. Fermentative DM losses in silage might be a result of CO_2_ production. CO_2_ is produced when lactic acid is converted to acetic acid, thus resulting in decreased LA with increased C_2_ [16,17]. This was consistent with the results of Kung Jr and Stanley [33], who found that a high degree of alcohol fermentation by yeasts during anaerobic fermentation in silage led to high rates of DM loss. To explain these observations, Suntara and Cherdthong [12] compared the characteristics of different yeast species, *S. cerevisiae*, *P. kudriavzevii* KKU20, and *C. tropicalis* KKU20, and indicated that *S. cerevisiae* could produce a higher amount of ethanol than the other species. In addition, according to Muck [34], DM loss might be caused by aerobic microorganisms in the silage. In the present study, aerobic bacteria increased at 21 days of RS ensilage as compared with 7 days, which may have resulted in DM loss. In this study, DM loss up to 8.08% occurred, which was lower than the range reported by Zimmer [35], who stated that DM loss can occur in a silage range of 7 to 40%. This might be because the amount of aerobic bacteria at 21 days did not cause much deterioration. Pholsen et al. [36] reported that aerobic bacteria should not exceed 10^6^ cfu/g FM in normal-quality silage; thus, when compared with the present study, aerobic bacteria were about 10^5^ cfu/g FM, and the ensiled RS was preserved well.

This experiment revealed that all yeast strains used to ferment RS could decrease fiber content in ensiled RS. Many years ago, it was discovered that yeast was capable of producing cellulase enzymes [37]. Previous research has indicated that some of the most important cellulase enzymes found in yeast are cellobiohydrolases (CBHs, EC 3.2.1.91), and that CBHs are potentially instrumental in hydrolysis of natural cellulose and breaking down the b-1,4-glycosidic bonds in fiber material [38]. In addition, Sarawan et al. [39] discovered that a new yeast strain named *C. konsanensis*, which is isolated from a plant in Thailand, can degrade cellulose crystals in test tubes. Suntara and Cherdthong [12] showed that *P. kudriavzevii* KKU20 and *C. tropicalis* KKU20 ruminal yeasts were capable of producing a cellulase enzyme at about 0.075 and 0.056 units/mL, respectively. Therefore, the reduction of NDF content by 2.76% in this study may have been caused by the cellulase enzymes produced by ruminal yeast.

### 4.2. Microorganism Populations after Ensiled RS

RS ensiled with all yeast strains showed decreased populations of LAB. Yeasts can survive in a wide pH range and under various anaerobic conditions. An optimum silage pH between 3.5 and 6.5 has been found for most yeast species, and some species can survive in a pH equal to or lower than 2.0 [40]. Under the anaerobic conditions in ensiled RS, yeast can convert sugar (as a carbon source) to ethanol through the alcoholic fermentation process. One molecule of glucose can be metabolized through a glycolysis pathway, and the oxidative processes can change the glycerol-3-phosphate into 1,3 bis-phosphoglycerate through glycerol-3-phosphate dehydrogenase. NAD^+^ was used as the coenzyme in a reoxidization mechanism in the reduction of acetaldehyde to ethanol [41]. This process provides two ATP molecules in a system, and the yeast growth rate will be stable or decrease [42].

Meanwhile, LAB require sugar (water-soluble carbohydrates) as a carbon source, generate energy for propagation during the early phase of the fermentation process, and provide lactic acid as the end product [40]. Consequently, when a significant amount of yeast occurs in ensiled RS, competition between yeast and LAB may occur. From the above reasons, our results were consistent with previous reports by Ávila et al. [43], who stated that the yeast populations and amount of ethanol production in silage are indicators for sugar utilization and lead to insufficient availability for LAB. Unfortunately, the present study did not measure ethanol production in ensiled RS; thus, this could not be confirmed. Nevertheless, previous studies demonstrated that *P. kudriavzevii* KKU20, *C. tropicalis* KKU20, and *S. cerevisiae* can produce ethanol amounts of about 44.2, 48.5, and 78.5 g/L, respectively [12]. Hence, it can be assumed that yeast competes with LAB for sugar in silage as a carbon source during the fermentation process, and influences the population of LAB. Although the present study showed that the control group had higher populations of LAB (6.90 Log10 cfu/g FM) compared to the RS ensiled with yeast (5.26 Log10 cfu/g FM), silage can be well preserved when LAB exceeds at least 5.0 Log10 cfu/g FM [17]; hence, ensiled RS was completely preserved in this study.

Yeast-fermented RS potentially showed decreased aerobic bacterial populations because that yeast competes for use of oxygen with aerobic bacteria in the silage. Pronk et al. [28] reported that when yeast had sufficient oxygen for oxidative phosphorylation, it produced maximum ATP to increase the population. However, when leaving the former stage and entering anaerobic conditions, yeast remains alive because it is able to switch from metabolism to alcoholic fermentation [42], whereas aerobic bacteria may be unable to propagate. This may have caused the aerobic bacteria to lose their competition with yeast during the initial stages, as they decreased by 30% compared to the yeast-supplemented group.

### 4.3. Fermentation Characteristics of Ensiled RS

This study revealed that 21 days of fermentation could increase the pH of ensiled RS from 4.08 (7 days) to 4.30 (21 days). Basically, silage enters a stable phase after 10–14 days of fermentation, and the pH does not alter until the silo is opened [44]. The pH values were not significant, but, interestingly, the pH was quite stable in the control group (4.09, 4.03, and 4.08 at 7, 14, and 21 days) while the yeast-supplemented group showed a pH increase (*p* = 0.07) with a long fermentation time (an average pH of 4.07, 4.18, and 4.37 at 7, 14, and 21 days). This result agreed with the reports of McDonald et al. [40], who reported that yeasts could raise the pH values in silages via assimilated lactic acid. Jianxin and Jun [45] recommend that silage pH could be used to categorize the silage quality, such as a pH of 3.4–3.8 for good silage, 3.9–4.1 for satisfactory silage, and 4.2–4.7 for average silage. This experiment showed that 21 days of fermentation may reduce the ensiled RS from satisfactory to average quality (pH 4.30). The decrease in lactic acid when ensiled RS was supplemented with yeast may correspond to the reduced LAB population and lead to a pH increase in silage. Furthermore, the low nutritional value of RS with a high fiber content (compared to green forage) could also be a result of higher pH. However, the amount of lactic acid in this study was still normal (20.53–26.14 g/kg DM). This aligned with the results of Jianxin and Jun [45], who suggested that lactic acid in silage should be in a range of 16–25 g/kg DM to yield good silage. The C_2_ concentration increased when the ensiled RS was supplemented with yeast, possibly because a proliferation of acetic acid bacteria appeared in the silage. Muck [33] reported that acetic acid bacteria can metabolize ethanol to provide C_2_ as an end product. According to this study, ethanol may be assumed to occur during fermentation. It was utilized by acetic acid bacteria, resulting in the accumulation of acetic acid in ensiled RS. The C_4_ concentration in silage ranged from 0.75 to 0.82 g/kg DM, which was similar to that reported by Muck [33]. C_4_ concentration in the silage fermentation process is an indicator of silage quality, and it should detectable at a low level in well-fermented silages. Muck [33] indicated that a C_4_ concentration of more than 5 g/kg DM in silage may reduce intake by animals, with evidence of substantial clostridial activity. The smell of silage contaminated with Clostridia is vomit-like, caused by higher butyric acid (BA) content. Therefore, besides the pungent odor, a major problem on the farm is refusal of silage intake by animals [16]. Additionally, NH_3_–N concentration in RS silage is used to evaluate silage quality, which is typically related to proteolytic Clostridia activity in degradation of protein [17]. Thus, a high concentration of NH_3_–N concentration in silage may indicate low silage quality. However, the present study revealed that there was unchanged NH_3_–N values in silage. It possibly is that the silage was well preserved with suitable pH, thus resulting in inhibition of proteolytic Clostridia activity.

### 4.4. Gas Production, In Vitro Digestibility, and Microbial Population

Yeast fermented with RS clearly showed more potential for improved in vitro gas production parameters than did RS without yeast inoculants. In particular, Crabtree-negative yeast increased cumulative gas production about 6.8% more than Crabtree-positive yeast at 96 h of incubation. The digestibility relates to gas production [46], and *P. kudriavzevii* KKU20 (Crabtree-negative yeast) showed great potential in this regard. The *P. kudriavzevii* KKU20 had outstanding in vitro digestibility when fermented with RS for 14 days. Compared to Crabtree-positive yeast, the cumulative gas production, IVDMD, and IVNDFD increased by 8.47%, 4.92%, and 6.75%, respectively. This may have occurred because Crabtree-negative ruminal yeast can produce more of the cellulase enzyme than Crabtree-positive yeast [12], thus increasing gas production and digestibility. The effect of Crabtree-negative yeast on increasing gas production was supported by studies from Wang et al. [1], who found that *C. tropicalis* KKU20 produced 3.03% more gas than did *S. cerevisiae*.

In addition, microbial populations in the rumen fluids are a significant factor affecting the increase in gas production and in vitro digestibility. Jayanegara et al. [47] reported that the decrease in bacterial and fungal populations in rumen fluids could reduce gas production and digestion potential. Therefore, any additive that can increase the microbial population in the rumen is necessary to enhance the in vitro gas parameter. This present study revealed that the ensiled RS showed increased bacterial populations and fungal zoospores by 11.06% and 10.6%, respectively. This agreed with the reports of Khampa et al. [2] who reported that fermented cassava shipped with *S. cerevisiae* increased the bacterial and fungal zoospore populations by 53.2% and 46.0%, respectively. Consistent with Koatdoke et al. [48], *S. cerevisiae* fermented with cassava pulp increased bacterial and fungal zoospore populations by 32.2% and 40.0%, respectively. In addition, Foiklang et al. [4] ensilaged RS with *S. cerevisiae*, and the result showed that in vitro true digestibility increased by 9.6% when compared with the no-yeast group. Chaucheyras-Durand et al. [49] stated that yeast favors the development and maturation of rumen, which contributes to the establishment of microbiota [50]. Yeast could provide rumen with biological stimulants, such as carbohydrates (manno-oligo saccharide), protein or amino acid chelates (B-complex), and minerals. These are necessary for microorganisms’ growth in the rumen [11]. However, no previous studies have compared the effectiveness of various types of yeast like this present study did. Interestingly, this study found that Crabtree-negative yeast showed a higher potential (compared to Crabtree-positive yeast) for stimulating bacterial populations and fungal zoospore by 3.34% and 3.8%, respectively. Thus, the enhancement of rumen microorganisms by Crabtree-negative yeast could help to improve feed digestion and increase gas production.

### 4.5. Ruminal Fermentation Products

RS ensilage with yeast had a positive effect on ruminal TVFAs. The high production of total VFA in the rumen relates to feeding digestibility and to the amount of microbial biomass in rumen fluids [51]. Moreover, some research has stated that increasing rumen microorganisms could increase the production of VFAs in the rumen [52]. Mutsvangwa et al. [53] found that adding *S. cerevisiae* increased the concentration of acetate and TVFAs in vitro and in vivo when compared to no treatment. Thus, using yeast clearly has a positive effect on the rumen environment. However, the present study found no different effects on TVFAs between Crabtree-negative and Crabtree-positive yeast treatments (86.55 and 87.11 mmol/L, respectively). Similarly, Wang et al. [1] found no difference in TVFAs between *C. tropicalis* (Crabtree negative) and *S. cerevisiae* (Crabtree-positive).

Our results showed that the C_3_ concentration increased after adding yeast to ensiled RS. However, both Crabtree-negative and Crabtree-positive yeasts were nearly equal regarding the C_3_ increase in the rumen. Yeast cells enriched with nutrients are adequate for supplying the growth of lactic-acid-utilizing bacteria (LUB) such as *Megaspaera elsdinii* and *Selemonas ruminantium*. Williams et al. [54] reported that yeast could stimulate the LUB population in the rumen, and LUB is the main way to produce C_3_ via the acrylate pathway [55]. Supporting this, Chung et al. [56] reported that yeast supplementation increased C_3_ yields and decreased the rumen’s C_2_:C_3_ ratio. LUB have a high ability to utilize lactic acid in the rumen [55,57]. C_3_ encourages a competitive pathway for H_2_ with other bacteria in the rumen [58] via the acyl-CoA dehydrogenase enzyme’s activity. Hydrogen is utilized and transformed into C_3,_ and finally provided to the animal [59].

## 5. Conclusions

In conclusion, RS ensilage with isolated Crabtree-negative ruminal yeasts could benefit feed digestion and in vitro gas production more that with Crabtree-positive yeast. However, no difference in ensilage quality, nutritive value, or microorganism composition appeared when Crabtree-negative ruminal yeasts and Crabtree-positive yeasts were used to treat RS. *P. kudriavzevii* KKU20, an isolated Crabtree-negative ruminal yeast used to treat RS, had the highest potential for increasing cumulative gas production and enhancing in vitro digestibility. This study recommends that keeping RS ensiled with yeast for 14 days could provide good silage quality. However, further evaluations of ruminal Crabtree-negative yeast (*P. kudriavzevii* KKU20) fermented with RS with in vivo experiments are required.

## Figures and Tables

**Table 1:** Chemical composition (g/kg) rice straw fermented with Crabtree-positive and -negative yeasts (DM basis).

Yeasts	Fermentation Days	Dry Matter g/kg	g/kg Dry Matter					
			Organic Matter	Ether Extract	Crude Protein	Neutral Detergent Fiber	Acid Detergent Fiber	Acid Detergent Lignin
Control	7	296.6	828.0	6.57	61.46	735.2	481.3	63.41
	14	289.7	859.0	6.79	60.43	722.1	480.3	62.88
	21	280.2	860.8	6.77	59.90	727.5	452.1	64.99
*P. kudriavzevii* KKU20	7	287.0	870.7	7.09	60.03	694.7	452.4	63.28
	14	283.3	880.2	5.97	61.37	704.7	493.6	66.00
	21	263.8	879.9	6.23	60.93	721.5	446.9	68.10
*C. tropicalis* KKEU20	7	286.7	848.9	6.35	60.10	697.6	473.5	65.60
	14	281.0	865.3	6.49	60.63	701.5	462.5	65.23
	21	259.3	862.2	7.13	60.97	711.4	469.8	62.60
*S. cerevisiae KKU20*	7	272.1	869.7	6.87	60.63	698.9	483.4	62.50
	14	268.0	867.1	6.67	60.90	716.0	460.1	65.63
	21	249.8	872.3	6.80	60.00	695.8	451.0	64.43
SEM		9.65	7.12	0.67	0.46	10.2	1.58	0.29
Comparison								
Yeast	Control	289.0 ^a^	849.1 ^c^	6.71	60.90	728.3 ^a^	4671.2	64.87
	*P. kudriavzevii* KKU20	277.8 ^ab^	872.3 ^a^	6.43	60.00	706.9 ^b^	464.3	65.79
	*C. tropicalis* KKU20	275.5 ^ab^	859.5 ^bc^	6.66	59.10	703.5 ^b^	468.6	64.48
	*S. cerevisiae*	263.2 ^b^	870.7 ^ab^	6.78	58.20	703.5 ^b^	464.8	64.19
Day	7	285.0 ^a^	854.9 ^b^	6.72	57.30	706.6	472.6	64.53
	14	280.7 ^a^	868.1 ^a^	6.48	56.40	711.1	474.1	64.94
	21	262.4 ^b^	869.3 ^a^	6.73	55.50	714.0	454.9	65.03
Interaction	Yeast × Day	ns	ns	ns	ns	ns	ns	ns
Orthogonal contrast								
Contrast 1	Control	289.0 ^a^	849.27	6.71	60.60	728.3 ^a^	471.2	63.76
	Yeast	272.9 ^b^	868.47	6.62	60.62	704.7 ^b^	465.9	64.82
Contrast 2	Yeast (−)	276.3	867.87	6.54	60.51	705.2	466.4	64.19
	Yeast (+)	263.2	869.67	6.78	60.67	703.6	464.8	65.14

SEM: standard error of the mean; ns: mean non-significant among treatment; ^a,b,c^ Means in the same row with different superscripts differ (*p* < 0.01, *p* < 0.05).

**Table 2:** Microbiological analysis of rice straw fermented with Crabtree-positive and -negative yeasts.

Strain	Ensilage Times (Day)	Microorganisms Log10 (cfu/g FM)				
		Yeast	Lactic Acid Bacteria	Coliform	Aerobic Bacteria	Mold
Control	7	5.89 ^d^	6.72	ND	6.52 ^ab^	ND
	14	4.53 ^e^	6.86	ND	5.53 ^abcd^	ND
	21	4.11 ^e^	7.15	ND	6.77 ^a^	ND
*P. kudriavzevii* KKU20	7	10.3 ^a^	5.12	ND	2.98 ^g^	ND
	14	9.44 ^a^	5.29	ND	3.54 ^fg^	ND
	21	7.89 ^bc^	5.22	ND	6.14 ^abc^	ND
*C. tropicalis* KKU20	7	9.85 ^a^	5.33	ND	3.77 ^efg^	ND
	14	9.05 ^ab^	5.37	ND	4.45 ^def^	ND
	21	7.34 ^c^	5.33	ND	5.01 ^cde^	ND
*S. cerevisiae*	7	10.1 ^a^	5.28	ND	2.90 ^g^	ND
	14	9.56 ^a^	5.13	ND	5.16 ^bcd^	ND
	21	7.65 ^c^	5.25	ND	5.52 ^abcd^	ND
SEM		0.21	0.15	ND	0.23	
Comparison						
Yeast	Control	4.84	6.90 ^a^	ND	6.27	ND
	*P. kudriavzevii* KKU20	9.01	5.21 ^b^	ND	4.22	ND
	*C. tropicalis* KKU20	8.57	5.34 ^b^	ND	4.41	ND
	*S. cerevisiae*	8.90	5.22 ^b^	ND	4.53	ND
Day	7	8.69	5.61	ND	4.04	ND
	14	7.73	5.66	ND	4.67	ND
	21	6.76	5.74	ND	5.86	ND
Interaction	Yeasts × Day	*	Ns	ND	*	ND
Orthogonal Contrast						
Contrast 1	Control	4.85 ^b^	6.90 ^a^	ND	6.27 ^a^	ND
	Yeast	9.03 ^a^	5.26 ^b^	ND	4.39 ^b^	ND
Contrast 2	Yeast (−)	8.98	5.28	ND	4.31	ND
	Yeast (+)	9.11	5.22	ND	4.53	ND

SEM: standard error of mean; ns, mean non-significant among treatment; ^a,b,c,e,f,g^ Means in the same row with different superscripts differ (*p* <0.01, *p* < 0.05); cfu/g FM: colony-forming unit per gram of fresh matter. ND: not detected; * indicates significant differences (*p* < 0.05).

**Table 3:** Acidity (pH), ammonia–nitrogen, and organic acid composition in rice straw fermented with Crabtree-negative and -positive yeasts.

Yeast	Ensilage Times (day)	pH	Ammonia Nitrogen	Organic Acid (g/kg Dry Matter)			
			(g/kgDM)	Lactic Acid	Acetic Acid	Propionic Acid	Butyric Acid
Control	7	4.09	1.60	26.1	4.36	ND	0.78
	14	4.03	1.82	23.9	4.11	ND	0.75
	21	4.08	3.01	25.9	4.32	ND	0.81
*P. kudriavzevii* KKU20	7	4.18	1.17	23.5	5.59	ND	0.80
	14	4.23	1.95	20.9	5.45	ND	0.84
	21	4.47	2.56	21.4	5.12	ND	0.79
*C. tropicalis* KKU20	7	4.02	1.66	22.7	5.25	ND	0.81
	14	4.14	1.78	20.5	5.16	ND	0.82
	21	4.35	2.21	23.2	5.03	ND	0.80
*S. cerevisiae*	7	4.04	1.59	22.4	5.32	ND	0.80
	14	4.17	1.99	24.1	5.40	ND	0.83
	21	4.31	2.15	21.9	5.35	ND	0.83
SEM		0.10	0.69	1.37	0.36		0.04
Comparison							
Yeast	Control	4.07	2.14	25.3 ^a^	4.26 ^b^	ND	0.78
	*P. kudriavzevii* KKU20	4.30	1.89	21.9 ^b^	5.38 ^a^	ND	0.81
	*C. tropicalis* KKU20	4.17	1.88	22.1 ^b^	5.14 ^a^	ND	0.81
	*S. cerevisiae*	4.17	1.91	22.8 ^b^	5.36 ^a^	ND	0.82
Day	7	4.08 ^b^	1.50	23.7	5.13	ND	0.80
	14	4.15 ^b^	1.88	22.4	5.03	ND	0.81
	21	4.30 ^a^	2.49	23.1	4.96	ND	0.81
Interaction	Yeasts × Day	Ns	ns	ns	ns		ns
Orthogonal Contrast							
Contrast 1	Control	4.07	2.14	25.3 ^a^	4.26 ^b^	ND	0.78
	Yeast	4.21	1.90	22.3 ^b^	5.29 ^a^	ND	0.81
Contrast 2	Yeast (+)	4.23	1.89	22.0	5.27	ND	0.81
	Yeast (−)	4.17	1.91	22.8	5.36	ND	0.82

SEM: standard error of mean; ns: non-significant among treatment; ^a,b^ Means in the same row with different superscripts differ (*p* < 0.01, *p* < 0.05); ND: not detected.

**Table 4:** Gas production kinetics and degradability of rice straw fermented with Crabtree-negative and -positive yeasts from in vitro incubation.

Strain	Ensilage Times (Day)	Gas Kinetics				Gas Volume	Degradability (g/kg Dry Matter)			
		a	b	c	a + b	96 h	IVDMD	IVOMD	IVNDFD	IVADFD
Control	7	4.44	103.6	0.052 ^a^	108.1	100.1	437.3 ^d^	607.5	699.3 ^b^	484.8
	14	4.90	110.0	0.050 ^ab^	114.9	112.8	571.5 ^c^	591.5	699.7 ^b^	465.5
	21	3.83	104.3	0.051 ^ab^	108.1	101.1	602.3 ^abc^	593.0	699.9 ^b^	463.3
*P. kudriavzevii* KKU20	7	5.55	114.1	0.048 ^b^	119.7	117.8	623.1 ^abc^	715.9	706.1 ^b^	516.6
	14	4.43	130.5	0.060 ^a^	134.9	130.5	639.8 ^a^	674.7	774.0 ^a^	493.2
	21	3.91	123.7	0.059 ^a^	127.6	122.5	608.4 ^abc^	676.5	696.3 ^b^	494.4
*C. tropicalis* KKU20	7	5.30	116.9	0.061 ^a^	122.2	115.4	619.3 ^abc^	670.2	715.0 ^b^	503.4
	14	4.87	119.4	0.060 ^a^	124.3	119.9	631.8 ^ab^	691.0	718.5 ^b^	505.4
	21	2.94	117.6	0.057 ^a^	120.5	118.2	579.7 ^bc^	675.2	732.4 ^b^	496.4
*S. cerevisiae*	7	0.96	105.7	0.051 ^ab^	106.6	106.3	630.7 ^ab^	696.2	716.5 ^b^	501.8
	14	2.40	120.0	0.048 ^ab^	122.4	120.3	609.8 ^abc^	681.7	725.7 ^b^	498.3
	21	3.22	115.3	0.049 ^ab^	118.5	114.8	617.0 ^abc^	679.0	727.3 ^b^	500.3
SEM		1.26	3.16	0.02	3.51	3.49	1.63	1.60	1.29	1.27
Comparison										
Yeast	Control	4.39	105.9 ^c^	0.051	110.3 ^b^	104.7 ^c^	537.0	597.3 ^b^	699.6	471.2 ^b^
	*P.kudriavzevii* KKU20	4.63	122.8 ^a^	0.055	127.4 ^a^	123.6 ^a^	623.8	689.1 ^a^	725.5	501.4 ^a^
	*C. tropicalis* KKU20	4.37	117.9 ^ab^	0.060	122.3 ^a^	117.8 ^ab^	610.2	678.8 ^a^	722.0	501.7 ^a^
	*S. cerevisiae*	2.20	113.6 ^b^	0.049	115.8 ^b^	113.8 ^b^	619.2	685.7 ^a^	723.2	500.1 ^a^
Day	7	4.06	110.1 ^c^	0.053	114.1 ^b^	109.9 ^b^	577.6	672.5	709.2	501.7
	14	4.15	119.9 ^a^	0.054	124.1 ^a^	120.9 ^a^	613.2	659.7	729.5	490.6
	21	3.48	115.2 ^b^	0.054	118.7 ^b^	114.1 ^b^	601.8	655.9	714.0	488.6
Interaction	Yeast × Day	ns	ns	**	Ns	ns	**	ns	*	ns
Orthogonal Contrast										
Contrast 1	Control	4.39	105.9 ^b^	0.051	110.3 ^b^	104.7 ^b^	53.7 ^b^	537.0 ^b^	597.3 ^b^	699.6 ^b^
	Yeast	3.73	118.1^a^	0.055	121.9 ^a^	118.4 ^a^	61.8 ^a^	617.7 ^a^	684.5 ^a^	723.5 ^a^
Contrast 2	Yeast (−)	4.50	120.4 ^a^	0.058 ^a^	124.9 ^a^	120.7 ^a^	61.7	617.0	683.9	723.7
	Yeast (+)	2.20	113.6 ^b^	0.049 ^b^	115.8 ^b^	113.8 ^b^	61.9	619.2	685.7	723.2

SEM: standard error of mean; ns: non-significant among treatment; ^a,b,c^ Means in the same row with different superscripts differ (*p* <0.01, *p* < 0.05); ** indicates significant differences at *p* < 0.01; * indicates significant differences at *p* < 0.05. Gas kinetics include: a: volume of gas produced from soluble fraction, b: volume of gas produced from insoluble fraction, c: gas production rate constant for insoluble fraction, a + b: potential extent of gas production; gas volume at 96 h after incubation (mL/0.5 g DM substrate). Degradability includes: IVDMD: in vitro dry matter digestibility, IVOMD: in vitro organic matter digestibility, IVNDFD: in vitro neutral detergent fiber digestibility, IVADFD: in vitro acid detergent digestibility fiber digestibility.

**Table 5:** Volatile fatty acid concentration of rice straw fermented with Crabtree-negative and -positive yeasts from in vitro incubation.

Yeasts	Ensilage Times (Day)	Total Volatile Fatty Acids (mmol/L)			Acetic Acid (mol/100 mol)			Propionic Acid (mol/100 mol)			Butyric Acid (mol/100 mol)		
		4 h	8 h	Mean	4 h	8 h	Mean	4 h	8 h	Mean	4 h	8 h	Mean
Control	7	68.8	80.2	74.5	72.6	70.9	72.1 ^ab^	16.7	17.6	16.7	10.7	11.5 ^a^	11.1 ^a^
	14	72.8	84.4	78.6	71.0	70.2	69.9 ^bc^	18.3	18.3	19.0	10.7	11.6 ^a^	11.1 ^a^
	21	70.4	81.7	76.0	73.4	75.6	74.9 ^a^	15.8	16.0	14.8	10.9	8.4 ^ed^	9.65 ^bc^
*P. kudriavzevii* KKU20	7	88.6	91.4	90.0	70.8	66.0	68.8 ^bc^	20.0	25.0	22.2	9.20	8.9 ^cd^	9.07 ^cd^
	14	89.7	89.3	89.5	68.6	68.8	68.7 ^bc^	21.5	24.2	22.9	9.85	7.08 ^e^	8.46 ^d^
	21	87.2	88.6	87.9	63.9	64.3	64.2 ^d^	26.3	24.8	25.3	9.86	10.9 ^ab^	10.4 ^ab^
*C. tropicalis* KKU20	7	88.6	90.5	84.4	71.8	65.3	70.2 ^bc^	18.4	24.7	21.0	9.85	9.6 ^cd^	9.88 ^bc^
	14	80.7	82.6	81.7	68.1	65.3	66.6 ^cd^	23.0	25.5	24.3	9.10	9.7 ^bcd^	9.16 ^cd^
	21	78.4	83.0	85.8	68.9	65.4	67.1 ^cd^	21.2	26.3	23.9	9.89	8.3 ^ed^	9.11 ^cd^
*S. cerevisiae*	7	86.3	90.5	88.4	68.4	65.0	66.8 ^cd^	22.0	26.0	23.3	9.53	9.4 ^cd^	9.61 ^bc^
	14	85.8	86.8	86.7	68.9	67.0	68.6 ^bc^	21.8	22.2	21.0	9.33	9.9 ^bc^	9.53 ^bc^
	21	83.9	88.5	86.2	69.0	66.7	67.8 ^cd^	21.3	24.9	22.3	9.63	9.3 ^cd^	9.49 ^bc^
SEM		1.96	2.55	1.58	2.47	1.46	1.27	2.67	1.76	1.2	0.26	0.40	0.29
Comparison													
Yeast	Control	70.64 ^c^	82.1 ^b^	76.4 ^c^	72.3	72.2 ^a^	72.3	16.9	17.3 ^b^	16.9 ^b^	10.8 ^a^	10.50	10.6
	*P. kudriavzevii* KKU20	88.52 ^a^	89.7 ^a^	89.1 ^a^	67.8	66.4 ^b^	67.2	22.6	24.6 ^a^	23.5 ^a^	9.64 ^b^	9.00	9.32
	*C. tropicalis* KKU20	82.6 ^b^	85.4 ^ab^	83.9 ^b^	69.6	65.3 ^b^	68.0	20.9	25.5 ^a^	23.0 ^a^	9.61 ^b^	9.19	9.38
	*S. cerevisiae*	85.3 ^ab^	88.6 ^a^	87.1 ^a^	68.8	66.3 ^b^	67.7	21.7	24.4 ^a^	22.2 ^a^	9.50 ^b^	9.55	9.55
Day	7	83.0	88.1	84.3	70.9	66.8	69.5	19.3	23.3	20.8	9.83	9.85	9.92
	14	82.3	85.8	84.1	69.2	67.8	68.4	21.2	22.5	21.8	9.74	9.57	9.57
	21	80.0	85.5	84.0	68.8	68.0	68.5	21.2	23.0	21.6	10.1	9.26	9.67
Interaction	Yeast × Day	ns	ns	ns	ns	ns	*	ns	ns	ns	ns	**	**
Orthogonal Contrast													
Contrast 1	Control	70.6 ^b^	82.06 ^b^	76.4 ^b^	72.3 ^a^	72.2 ^a^	72.3 ^a^	16.9 ^b^	17.3 ^b^	16.8 ^b^	10.8 ^a^	10.5 ^a^	10.6 ^a^
	Yeast	85.5 ^a^	87.91 ^a^	86.7 ^a^	68.7 ^b^	65.9 ^b^	67.6 ^b^	21.7 ^a^	24.8 ^a^	22.9 ^a^	9.58 ^b^	9.25 ^b^	9.42 ^b^
Contrast 2	Yeast (−)	85.5	87.6	86.55	68.7	65.8	67.6	21.7	25.1	23.3	9.63	9.10	9.35
	Yeast (+)	85.3	88.6	87.11	68.8	66.3	67.7	21.7	24.4	22.2	9.50	9.55	9.55

SEM: standard error of mean; ns: non-significant among treatmentS; ^a,b,c,d^ Means in the same row with different superscripts differ (*p* < 0.01, *p* < 0.05); ** indicates significant differences at *p* < 0.01; * indicates significant differences at *p* < 0.05.

**Table 6:** Microbial populations in rumen fluids from rice straw fermented with Crabtree-negative and -positive yeasts from in vitro incubation.

Yeasts	Fermentation Days	Bacteria (cell/mL)			Protozoa (cell/mL)			Fungi Zoospore (cell/mL)		
		4 h	8 h	Mean	4 h	8 h	Mean	4 h	8 h	Mean
Control	7	7.32	7.34 ^d^	7.33 ^e^	5.86	6.09	5.97	5.80	6.06	5.93
	14	7.09	7.38 ^d^	7.23 ^ef^	5.80	6.29	6.05	5.86	6.16	6.01
	21	6.87	7.35 ^d^	7.11 ^f^	5.70	6.06	5.88	5.90	5.96	5.93
*P. kudriavzevii* KKU20	7	8.16	8.29 ^a^	8.22 ^a^	5.80	6.06	5.93	6.50	6.84	6.67
	14	8.12	8.27 ^a^	8.19 ^a^	5.58	5.73	5.66	6.36	6.72	6.54
	21	7.93	8.22 ^a^	8.08 ^ab^	5.63	6.16	5.90	6.30	6.84	6.57
*C. tropicalis* KKU20	7	8.08	8.24 ^a^	8.16 ^ab^	5.83	6.00	5.92	6.56	6.88	6.72
	14	8.00	8.26 ^a^	8.13 ^ab^	5.70	6.09	5.90	6.52	6.71	6.61
	21	7.80	8.12 ^a^	7.95 ^bc^	5.80	5.60	5.70	6.40	6.54	6.47
*S. cerevisiae*	7	8.13	8.28 ^a^	8.21 ^a^	5.90	5.60	5.75	6.46	6.49	6.48
	14	7.76	7.89 ^b^	7.82 ^c^	5.60	5.57	5.58	6.50	6.60	6.55
	21	7.43	7.61 ^c^	7.52 ^d^	5.33	5.37	5.35	6.47	6.41	6.44
SEM		0.12	0.06	0.06	0.14	0.16	0.13	0.15	0.12	0.10
Comparison										
Yeast	Control	7.09 ^c^	7.36	7.23	5.79	6.15 ^a^	5.97	5.85 ^b^	6.06 ^c^	5.96 ^b^
	*P. kudriavzevii* KKU20	8.07 ^a^	8.26	8.17	5.67	5.98 ^a^	5.83	6.38 ^a^	6.79 ^a^	6.59 ^a^
	*C. tropicalis* KKU20	7.96 ^ab^	8.21	8.08	5.78	5.89 ^a^	5.84	6.49 ^a^	6.70 ^a^	6.60 ^a^
	*S. cerevisiae*	7.77 ^b^	7.93	7.85	5.61	5.51 ^b^	5.56	6.47 ^a^	6.50 ^b^	6.49 ^a^
Day	7	7.92 ^a^	8.04	7.98	5.85	5.94	5.89	6.33	6.57	6.45
	14	7.74 ^a^	7.95	7.85	5.67	5.92	5.80	6.31	6.55	6.43
	21	7.50 ^b^	7.83	7.67	5.62	5.80	5.71	6.27	6.44	6.35
Interaction	Yeast × Day	ns	**	**	ns	ns	ns	ns	ns	ns
Orthogonal contrast										
Contrast 1	Control	7.09 ^b^	7.16 ^b^	7.23 ^b^	5.79	6.15 ^a^	5.97 ^a^	5.85 ^b^	6.06 ^b^	5.96 ^b^
	Yeast	7.93 ^a^	7.93 ^a^	8.03 ^a^	5.69	5.79 ^b^	5.74 ^b^	6.45 ^a^	6.67 ^a^	6.56 ^a^
Contrast 2	Yeast (−)	8.01 ^a^	8.03 ^a^	8.12 ^a^	5.72	5.94 ^a^	5.83 ^a^	6.44	6.75 ^a^	6.60
	Yeast (+)	7.77 ^b^	7.73 ^b^	7.85 ^b^	5.61	5.51 ^b^	5.56 ^b^	6.47	6.50b	6.49

SEM: standard error of mean; ns: non-significant among treatments; ^a,b,c,d,e,f^ Means in the same row with different superscripts differ (*p* < 0.01, *p* < 0.05); ** indicates significant differences at *p* < 0.01.
